# Advancements in Bone Replacement Techniques–Potential Uses After Maxillary and Mandibular Resections Due to Medication-Related Osteonecrosis of the Jaw (MRONJ)

**DOI:** 10.3390/cells14020145

**Published:** 2025-01-20

**Authors:** Judit Bovari-Biri, Judith A Miskei, Zsanett Kover, Alexandra Steinerbrunner-Nagy, Kinga Kardos, Peter Maroti, Judit E Pongracz

**Affiliations:** 1 Department of Pharmaceutical Biotechnology, Faculty of Pharmacy, University of Pecs, 7624 Pecs, Hungary; bovari.judit@pte.hu (J.B.-B.); steinerbrunner.alexandra@pte.hu (A.S.-N.); 2 Department of Maxillo-Facial Surgery, Clinical Centre, The Medical School, University of Pecs, 7624 Pecs, Hungary; judith.miskei@aok.pte.hu (J.A.M.); kover.zsanett@pte.hu (Z.K.); 3 3D Printing and Visualization Centre, University of Pecs, 7624 Pecs, Hungary; kardos.kinga@pte.hu (K.K.); peter.maroti@aok.pte.hu (P.M.); 4 Medical Skills Education and Innovation Centre, Medical School, University of Pecs, 7624 Pecs, Hungary

**Keywords:** MRONJ, bone scaffolds, cellularization, stimulation of bone differentiation

## Abstract

Maxillofacial bone defects can have a profound impact on both facial function and aesthetics. While various biomaterial scaffolds have shown promise in addressing these challenges, regenerating bone in this region remains complex due to its irregular shape, intricate structure, and differing cellular origins compared to other bones in the human body. Moreover, the significant and variable mechanical loads placed on the maxillofacial bones add further complexity, especially in cases of difficult-to-treat medical conditions. This review provides a brief overview of medication-related osteonecrosis of the jaw (MRONJ), highlighting the medication-induced adverse reactions and the associated clinical challenges in treating this condition. The purpose of this manuscript is to emphasize the role of biotechnology and tissue engineering technologies in therapy. By using scaffold materials and biofactors in combination with autologous cells, innovative solutions are explored for the repair of damaged facial bones. The ongoing search for effective scaffolds that can address these challenges and improve in vitro bone preparation for subsequent regeneration in the maxillofacial region remains critical. The primary purpose of this review is to spotlight current research trends and novel approaches in this area.

## 1. Introduction to Medication-Related Osteonecrosis of the Jaw

The regeneration of medication-related osteonecrosis of the jaw (MRONJ) is a complex issue that requires the restoration of specific tissue microenvironments and, potentially, bone replacement. When considering treatment options, it is essential to evaluate multiple factors based on the clinical stage of the condition. A patient-centered, multidisciplinary approach is crucial for selecting the most appropriate therapy. This involves a patient-focused perspective to identify the best treatment based on the pathological stage, risk factors, and the most effective therapeutic methods aimed at improving their quality of life. This review aims to summarize the main causes of the disease, the biological background of the affected skeletal area, the novel developments in biotechnology and material science, the molecular background of bone development that can improve understanding MRONJ, and advances in available therapies involving tissue engineering. The biological characteristics of facial bones leading to MRONJ need fast-developing biotechnological methodologies and material developments with the aim of developing novel therapeutic pathways.

### 1.1. Epidemiology of MRONJ

Maxillofacial bone defects can result from congenital malformations, trauma, tumors, and inflammation, leading to altered jaw function and aesthetics. A less-known reason for maxillofacial bone surgery is MRONJ or “medication-related osteonecrosis of the jaw”, first reported in 2003 [1]. The epidemiology of MRONJ is multifaceted and varies significantly depending on the specific factors considered in each research study. Understanding these variations is crucial for developing effective prevention and treatment strategies. A study in Japan compared the incidence rate of MRONJ in nearly three million patients treated for osteoporosis and cancers [2]. The authors of the study also examined the impact of oral hygiene, gender, age, cancer type, and geolocation on the occurrence of MRONJ, suggesting that not only drugs but also microbial presence in the oral cavity play a significant role. More patients treated for cancer are affected by MRONJ than those treated for osteoporosis, with an approximate ratio of 1 patient with osteoporosis to 25 patients with cancer. The overall percentage of MRONJ patients remained under two percent among the total patient population examined. Recent studies show that the induction of MRONJ varies significantly based on the type of drug used in therapy, its dose, and treatment duration, ranging from 0.5% to 18%. Overall, however, the incidence of MRONJ in oncology patients remains below 5% [3]. There is still no straightforward conclusion regarding MRONJ frequency, as it depends on how many factors are taken into consideration. Studies indicate that MRONJ frequency ranges from very rare (less than 1 in 10,000) to common (1 in 100 or more) [4].

However, the condition of drug-induced osteonecrosis presents a significant clinical challenge. It involves painful exposure of the bone in the mandible or maxilla that does not necessarily respond to medical treatment. Additionally, there are currently no reliable predictive factors at the individual level to inform healthcare professionals or patients about the possibility of experiencing such adverse reactions to therapy. A lack of information can lead to severe clinical consequences and restrict treatment options. The development of effective treatments for a disease is unlikely if there is insufficient understanding of its underlying causes, particularly when these causes appear to be associated with specific medications. Understanding the relationship between the disease and these drugs is crucial for identifying potential therapeutic strategies and ensuring that treatments are both safe and effective.

### 1.2. Medications Triggering MRONJ

There are two main categories of pharmacological agents that can lead to osteonecrosis of the jaw: antiresorptive agents and antiangiogenic drugs. The antiresorptive group includes bisphosphonates (BPs) and receptor activators of nuclear factor kappa-B ligand (RANKL) inhibitors. In contrast, antiangiogenic agents include several medications that affect various signaling pathways. A summary of the medications that can trigger MRONJ is provided in Table 1.

Antiresorptive agents like bisphosphonates (zoledronic acid, alendronate, ibandronate, neridronate, etc.) bind to a mineral, hydroxyapatite. They incorporate into active bone remodeling sites in diseases characterized by accelerated skeletal turnover. Bisphosphonates can inhibit calcification and hydroxyapatite breakdown, thus efficiently suppressing bone resorption [5]. This essential property of bisphosphonate drugs has led to their clinical application. Recently, it has been suggested that bisphosphonates also act by restricting both osteoblast and osteocyte apoptosis [6,7]. Based on the pharmacological features of all bisphosphonates, they have become the primary therapy for skeletal disorders characterized by abnormal or imbalanced skeletal remodeling due to increased bone resorption by osteoclasts [8]. The other antiresorptive agent, the RANKL inhibitor denosumab, was developed for the treatment of osteoporosis for those with a high risk of fractures [9]. Unlike bisphosphonates, denosumab does not become integrated into the bone tissue network. Instead, this monoclonal antibody binds to RANKL in the extracellular fluids and blocks osteoclast development, decreasing bone resorption and increasing bone density [10].

Antiangiogenic medications act via inhibition of blood vessel formation by blocking angiogenesis. Based on their mechanism of action, antiangiogenic agents can be divided into three subgroups: monoclonal antibodies (e.g., bevacizumab), vascular endothelial growth factor (VEGF) Trap molecules (e.g., aflibercept) [11], and tyrosine kinase inhibitors (e.g., sunitinib, cabozantinib, sorafenib) [12]. Furthermore, inhibitors of the mammalian target of rapamycin (mTOR) signaling (rapamycin) also show antiangiogenic effects by decreasing the production of VEGF and platelet-derived growth factors (PDGF) [13].

Several drugs can trigger medication-related osteonecrosis of the jaw (MRONJ), and the chosen treatment approach depends not only on the specific medication but also on local factors such as inflammation from conditions like periodontitis or additional infections. The severity and stages of MRONJ play a crucial role in determining treatment strategies. In the early stages of the disease, conservative treatments may be effective. However, for more advanced cases, more aggressive surgical interventions are necessary. In these advanced stages, where the size of the lesion is increasing, tissue engineering (TE) methodologies, including the use of hydrogels and scaffolds, are often employed in clinical applications. Ultimately, treatment decisions should be individualized, with a careful consideration of whether to pursue conservative, invasive, or specially designed TE treatments based on the specifics of each case.

### 1.3. Stages of MRONJ

The combined use of antiangiogenic and antiresorptive agents substantially elevates the risk of MRONJ [14]. The main goal of using the medications listed in Table 1 is to strengthen bones weakened due to osteoporosis or bone metastasis. While these medications can improve bone strength in most parts of the body, significant necrosis in the maxilla and mandible can occur within months of starting treatment. The reasons for this phenomenon are largely unknown, but one possible clue is the fact that the embryonic developmental origins of the facial bones differ from those of the long bones [15]. To enhance understanding of outcomes in bone resorption and improve the chances of suitable treatment options, staging of medication-related osteonecrosis of the jaw (MRONJ) has become crucial. The staging of this medication-induced adverse reaction is summarized in Table 2.

### 1.4. Current Treatment Strategies in MRONJ

**Figure 1:**
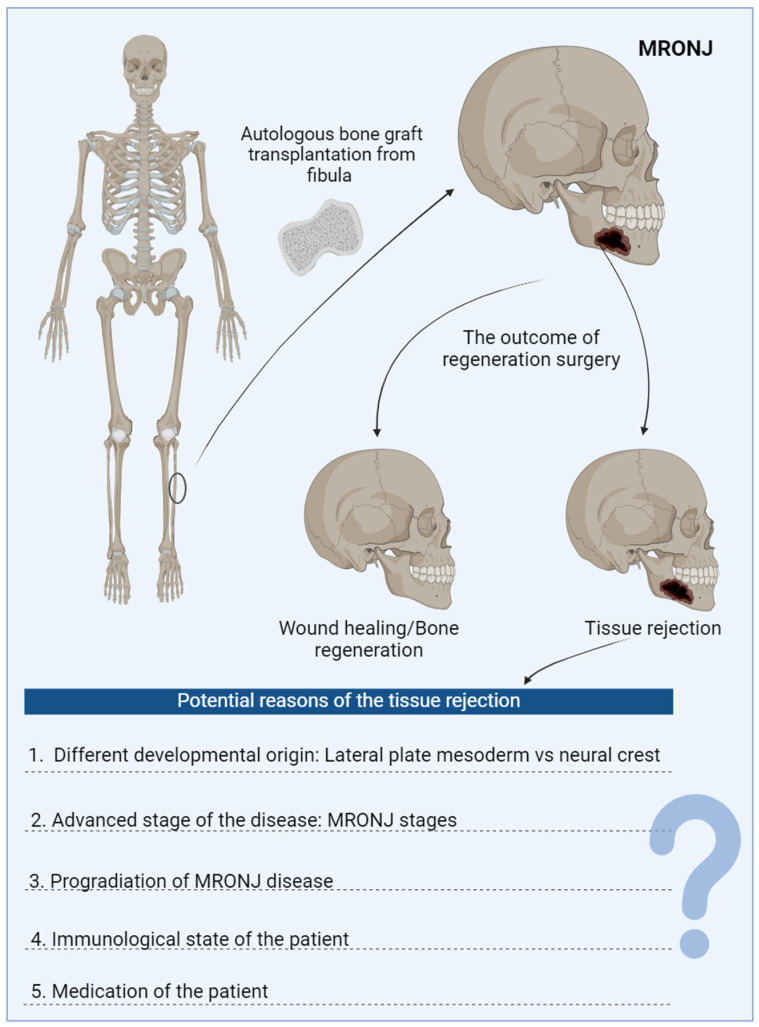
Autologous bone transplantation is a treatment option for managing medication-related osteonecrosis of the jaw (MRONJ). The accompanying figure illustrates the classic approach: using autologous bone from the patient, primarily taken from the long bone fibula. The healing process following this type of surgery can be lengthy and is not always successful. Several factors may contribute to the rejection of the graft. These factors, which are presented in the graph, warrant further investigation.

The identification of cortical destruction indicative of osteonecrosis can be achieved by performing a panoramic X-ray or computer tomography (CT) and CBCT (cone beam CT) scans. Despite the seemingly easy diagnosis of MRONJ by appearance, the pathophysiology of MRONJ has recently been identified as multifactorial and involves inhibition of bone remodeling, angiogenesis, inflammation, infection, immune dysfunction, and genetic factors. When a combination of these factors occurs, there is a high likelihood of developing MRONJ. Currently, however, there are no finalized guidelines for medical and/or surgical treatment for MRONJ. MRONJ may not show symptoms for extended periods. Symptoms typically manifest alongside physical signs, although pain may precede any visible indications. In the advanced stages, MRONJ commonly presents with pain and the discharge of pus from the exposed bone in the mandible or the maxilla. The teeth and gums may also be affected, and fistulas may form intra- or extraorally. Once established, MRONJ is challenging to treat. The treatment typically involves removal of the affected tissue, therapy with antibiotics, and antibacterial oral rinses (e.g., chlorhexidine). Although surgical resection of the affected area is not the initial treatment, it can become inevitable to restore facial symmetry and oral function. This condition severely affects a patient’s quality of life. If surgery involves a larger area, autologous bone replacement is a viable option as part of the treatment strategy (Figure 1). Even in healthy tissue, successful bone development or repair relies on the interplay of stem cells, growth, and other factors of the tissue microenvironment. When large bone defects occur, bone grafts or substitutes may be necessary. While autografts have been the gold standard, they are not always available. In addition, suitable donor allografts can also be used and show similar success rates [18]. Where allo- or autografts are not available, tissue engineering has become a viable option in recent years. Using α-tricalcium phosphate (α-TCP)—which with various additives is a self-setting component of bone cements, biodegradable bioceramics, and composites for repairing bone with or without mesenchymal stem cells (MSC)—can overcome the drawbacks of traditional bone grafts [19]. As reconstructing bone in the maxillofacial region poses unique challenges due to its complex structure, exposure to extensive mechanical stress, and the unique origins of the maxillofacial bones [20], a novel approach might be necessary to regenerate the tissue area affected by necrosis. Hence, tissue engineering has started to focus on developing fast and personalized bone tissue engineering in maxillofacial regions using ongoing clinical research for maxillofacial bone repair.

## 2. The Uniqueness of Maxillofacial Bones and Its Impact on Bone Tissue Engineering

The field of tissue engineering for maxillofacial bone replacement is well established. Nevertheless, tissue engineering still encounters several challenges that motivate researchers to explore this area more thoroughly. One major issue is that not all sources of autologous bones are suitable for successful surgical applications, which triggered further investigation. Moreover, comprehending the internal structure of the jaw and identifying the most suitable materials for bone substitution has also proven to be a challenging task. Tissue engineering and regenerative medicine aim to offer effective maxillofacial bone regeneration solutions, where continually improved biomaterial scaffolds play a significant role. For the effective development of maxillofacial bones with personalized characteristics, tissue engineering must extend beyond merely printing scaffolds. The design of the scaffold is crucial for supporting and integrating newly formed bone. Key factors include the choice of material, shape, pore size, and the incorporation of biomaterials that promote cellular migration and growth. Additionally, the use of hydrogels for the delivery of growth factors and drugs plays a significant role in creating an environment where stem cells can thrive and integrate.

### 2.1. Unique Origin of Maxillofacial Bones

Several studies have indicated that the bioengineering of bone tissue encompasses both the osteogenesis of stem cells and angiogenesis to support tissue development [21,22]. Undoubtedly, the source of stem cells could significantly influence bone healing, which is frequently disregarded in practical applications, and it is widely believed that stem cells have no imprinting and can form any kind of tissue. Influencing bone formation by stimulating directed stem cell migration and differentiation in a well-identified skeletal area is potentially one of the best examples.

**Figure 2:**
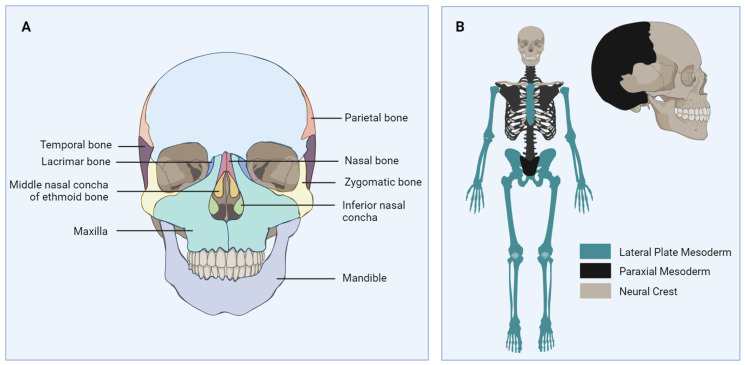
(**A**) Bones of the human facial skeleton (temporal bone (2); parietal bone (2); lacrimal bone (2); zygomatic bone (2); maxilla (2); mandible (1); interior nasal concha (2); vomer (1); palatine (2)). (**B**) Embryonic origin of the human skeleton. While the long bones originate from the lateral plate mesoderm, the human facial skeleton is from the neural crest.

The human facial skeleton is formed by fourteen bones (Figure 2A). The mandible and vomer are standalone bones in the maxillofacial complex. Any parts of the facial skeleton originate from neural crest stem cells.

Consequently, as the maxilla and mandible are also a part of the human facial skeleton, they too originate from the neural crest, while long bones come from the lateral plate mesoderm (Figure 2B) [15,23]. As it has been observed that bone marrow mesenchymal stem cells (BMSCs) from different embryonic origins may have specific preferences for different sites, understanding the development and difficult treatment of MRONJ might also be explained by such differences, especially as evidence was found that a “positional memory” exists [24] in skeletal stem cells, affecting their behavior when transplanted to different locations. Consequently, the origin of the MSCs must be selected carefully and positioned to the correct area, as neural crest-derived progenitors are more likely to repair the mandible, while mesoderm-derived progenitors preferentially repair, e.g., the tibia. Based on studies of mesoderm-derived progenitors, no effective differentiation was detected in osteoblasts for a mandible defect [25,26].

The exceptional osteogenic ability of orofacial BMSCs is well documented [27,28]. Therefore, BMSCs are excellent for rapid bone formation. To design scaffolds for therapeutic intervention in MRONJ, there are key steps to follow for a successful maxillofacial bone design that lead to regeneration of the damaged area:

(1) The location of the bone defect must be carefully determined to design the pore size and network, which can support cellular maintenance;
(2) The preference is a degradable scaffold, where the degradation rate is predesigned and coordinated with bone formation;
(3) To make the maxillofacial bone regeneration successful, MSCs are needed.

The ossification of maxillofacial bones goes through intramembranous ossification [29], with distinct molecular signals. Additionally, different molecular signals are involved in the processes of bone formation based on the mode of ossification using a distinct order of signal activation. For example, collagen type 1 alpha 1 chain (Col1a1) is responsible for the primary extracellular matrix of bone, whereas collagen type 2 alpha 1 chain (Col2a1) is essential for the cartilaginous template. Additionally, transcription factor SRY-Box Transcription Factor 9 (Sox9) plays a critical role in the differentiation of chondrocytes and the formation of cartilage. Meanwhile, WNT/β-catenin signaling promotes the differentiation of osteoblasts and inhibits the formation of chondrocytes [30].

To aid the development of viable, vascularized bone tissue and avoid avascular necrosis and core degradation, a combination of bone morphogenic protein-2 (BMP-2), VEGF, and fibroblast growth factors (FGF) (Table 3 [30,31,32,33,34,35,36,37,38,39]) are mixed into hydrogels (S-gelatin) and/or bound to nanobeads to fill up the pores of the scaffold and guide the required cell types to the expected scaffold area where they promote their differentiation [40,41]. This technique accelerates bone differentiation and vascularization, as discussed below.

**Figure 3:**
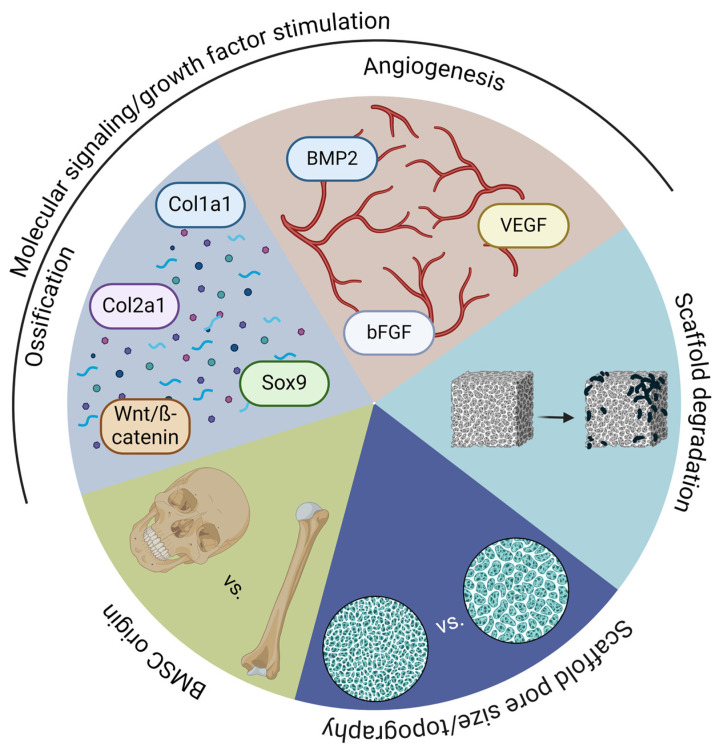
Summary figure of the crucial points of bone tissue engineering for clinical applications. The figure illustrates the various factors that can influence bone tissue regeneration: 1. The source of isolated stem cells plays a significant role in the regeneration process of maxillofacial bone. Stem cells derived from facial tissues are preferred over those from long bones, as they share a similar developmental origin. 2. The size and geometry of pores in scaffolds can affect their porosity, resistance to masticatory forces, and ability to promote bone ingrowth. 3. Scaffold degradation must be synchronized with accelerated tissue formation. Additionally, the degradation rate can be used to control the release of drugs, growth factors, and biomolecules. 4. Biomolecules that have crucial developmental roles can facilitate vascularization, cell differentiation, and the production of extracellular matrix (collagen type 1 alpha 1 chain (Col1a1), collagen type 2 alpha 1 chain (Col2a1), SRY-Box Transcription Factor 9 (Sox9), bone morphogenic protein (BMP), vascular endothelial growth factor (VEGF), fibroblast growth factor (FGF)).

While it appears that all the factors are well known and studied, especially the embryonic factors that lead to bone formation (Figure 3), the understanding of the maxillofacial bone healing process is incomplete.

### 2.2. Tissue Engineering Approach to Create Scaffolds for Maxillofacial Bones

To develop maxillofacial bones with personalized features, tissue engineering technologies must create appropriate scaffold architectures. The scaffold plays a crucial role in shaping the newly formed bone and restoring damaged facial features as well as physiological functions.

Additive manufacturing technology has significantly transformed the field of biomedicine in recent decades, allowing additional options for bone reconstructive surgery. Three-dimensional printing technology provides rapid and cost-effective prototyping and fabrication of scaffolds to be used in biomedical applications. Patient-specific implants, surgical guides, and anatomical or pathology models can be sufficiently produced and printed if needed [42,43]. The original printing technologies used a great variety of raw materials, including polymers, composites, metals, alloys, or ceramics, depending on the specific target application. All additive manufacturing solutions create objects “layer-by-layer” [44] to reduce material waste and production time and to create intricately detailed structures.

In tissue engineering, several additive manufacturing technologies are utilized for scaffold design and production, including fused deposition modelling (FDM), selective laser sintering (SLS), stereolithography (SLA), as well as 3D-bioplotter printing, direct 3D printing, and electrospinning methods [45]. In regenerative medicine, particularly in bone tissue fabrication, the most frequently used technologies involve ceramic-, hydrogel-, and polyester-based solutions [46], To create scaffolds for maxillofacial regeneration, the 3D-printed structures must accurately replicate the desired tissue morphology. Additionally, they should be capable of delivering drugs and biomolecules. This is essential for promoting the infiltration and differentiation of mesenchymal stem cells (MSCs), enhancing vascularization, and inhibiting bacterial growth. In cases where only small areas are affected by necrosis, injectable materials, such as in situ gelatinizing [47] and thermal-sensitive hydrogel scaffolds, are more appropriate for filling irregular bone defects [47,48], as they allow for controllable magnesium ion release or biomolecule delivery to enhance bone formation [49].

### 2.3. Scaffold Materials Suitable for Maxillofacial Bone Repair

Combination use of PLGA (poly(lactic-co-glycolic acid)), PCL (polycaprolactone), and TCP (tricalcium phosphate) are frequently used scaffold materials. Medical-grade PCL is a particularly promising candidate in maxillofacial bone regeneration [50]. To find the ideal material, a combination of PCL with hydroxyapatite (HA) or TCP [51,52,53,54] is also tested with 3D-printing technology to treat challenging zygomaticomaxillary defects with success [51,55]. PLGA can also be combined with HA, TCP, and TPU (thermoplastic polyurethane) [56]. Combining PCL, PLGA, and HA results in excellent mechanical properties and promotes cell attachment and proliferation, making it an advantageous material for 3D-printed bone tissue constructs [57]. PLGA/PCL electrospinning scaffold can be combined with various metal-like silver nanoparticles to reduce infection or inflammation following surgery [58,59,60] (Table 4 [50,51,52,53,54,55,56,57,58,59,60,61]).

While in vitro laboratory studies and in vivo animal testing demonstrate potential for various scaffolds, clinical studies on biomaterials used to repair medication-induced maxillofacial bone defects remain limited. The limitation of clinically applicable research results is not surprising. A wide array of safe and reliable clinical studies would be needed to identify the most suitable biomaterials, focusing on the appropriate pore sizes and shapes for effectively addressing individually sized maxillofacial defects at macro-, micro-, and nanoscale levels. Changes at the macroscale can accommodate the different needs of various parts of the maxillofacial bone structure [62]. At the same time, modifications at the micro/nanoscales can control the biological activity of attached cells and microscopic biomineralization [63,64]. For example, a PLGA/PCL scaffold is composed of three layers: a surface layer containing chlorhexidine, an opposing surface layer containing β-TCP, and a pure PLGA/PCL middle layer. This scaffold integrates the osteoconductive properties of β-TCP and the antibacterial function of chlorhexidine, with the middle layer enhancing its mechanical strength, making it particularly suitable for periodontal regeneration [65]. Another example is a biphasic PCL scaffold with a core–shell structure and a 3D-printed β-TCP scaffold with bimodal pore geometry used in load-bearing mandibular regions [53,66]. The complex 3D grid formed by the trabeculae is designed to withstand the forces generated during chewing. This structure is influenced by its spatial microstructures. Additionally, the nano-topography of these structures can affect how immune cells respond, which is particularly important during inflammatory conditions in the oral cavity [67,68]. These modifications at different scales can replicate natural structures and work together to meet the future’s diverse maxillofacial scaffolding needs.

### 2.4. Pore Size and Geometry

The bone tissue engineering scaffolds need to create a highly porous space, where the pores form an interconnected network. The design of the pore network is essential for sufficient cell seeding, supported and guided cell migration, and connection to the vasculature for sufficient nutrient and metabolic waste transport. To create tissue ingrowth for osteogenesis during the first 4 weeks after implantation, all pore size-, geometry-, and osteogenesis-stimulating factors must be present to promote bone tissue ingrowth, bearing strength, and soft tissue invasion.

**Figure 4:**
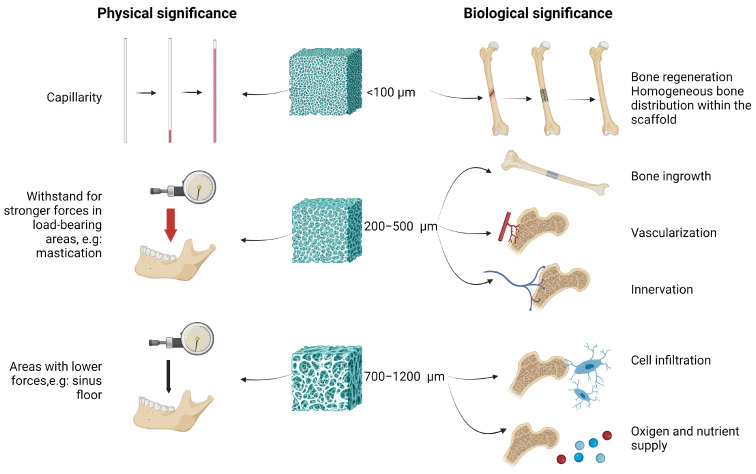
Graphical representation of physical and biological significance of various pore sizes. Graphical representation of physical and biological significance of various pore sizes. <100 µm: Due to the high capillary effect of the small pore size (results in better nutrient supply), the bone regeneration process can be improved; 200–500 µm: ideal for improving resilience to mastication forces, bone ingrowth, vascularization, and innervation; 700–1200 µm: useful in areas with lower forces (e.g., sinus floor) and can ensure optimal cell infiltration and oxygen/nutrient supply.

Generally, to create the optimal pore size, the following is considered (Figure 4):

(1) Porosity of 70–90% with pore sizes ranging from 300–500 μm is recommended to promote bone ingrowth, vascularization, and innervation [69,70,71,72].
(2) Recent studies indicate that the optimal pore dimensions for maxilla–mandibular scaffolds range from 700 to 1200 μm. However, the ideal pore size should be tailored to the specific location of the bone within the maxillofacial region. For repairing mandibular defects, a pore dimension of 600 μm is recommended as the most beneficial. Larger pore sizes can enhance the supply of nutrients and oxygen, which promotes osteogenesis. Nevertheless, when pore dimensions exceed a certain size, the levels of nutrient and oxygen supply can become saturated. Additionally, excessively large pore dimensions may hinder the ability to create interconnections within the bone [73,74].
(3) Pore size should be tailored to different forces and cell types in specific surgical locations. Various pore sizes and shapes are required in different regions to promote cellular infiltration. For instance, a pore size of 490 μm is more appropriate for load-bearing areas, such as the lateral mandible, while a pore size of approximately 750 μm may enhance cell infiltration in regions with lower forces, such as the sinus floor [75,76].
(4) Smaller pore sizes (200–300 μm) are less likely to cause soft tissue invasion, preventing fibrous tissue penetration. When constructing scaffolds with larger pores, barrier membranes may be necessary to achieve superior bone formation [67].

Scaffold macropores (greater than 100 μm) are essential for promoting bone ingrowth and vascularization, while microporosity (less than 20 μm) plays a vital role in bone growth at sites of injury. However, when microporous scaffolds are immersed in phosphate-buffered saline to eliminate active capillary forces induced by the micropores before implantation, the uniformity of bone distribution can be compromised. Despite this, the method shows promise for treating large, load-bearing bone defects [77,78].

### 2.5. Mechanical Properties of the Scaffold

Scaffolds for bone engineering must possess mechanical properties that align with the tissues at the implantation site to offer sufficient mechanical support and prevent excessive deformation [79,80]. Maxillofacial bone exhibits a higher remodeling rate and lower mineralization and mass density than the femur, primarily due to larger osteocyte lacunae [25,81]. Consequently, the low levels of bone mineralization result in increased fracture toughness, which should be considered when designing scaffolds for maxillofacial bone regeneration [82,83,84]. The different maxillofacial regions vary in mechanical performance:

(1) The anterior mandible’s trabecular bone has higher density and increased elastic modulus and compressive strength compared to other regions [85].
(2) When dealing with large bone defects across various maxillofacial regions that must withstand masticatory forces, scaffold materials and pore size should be tailored to meet the specific mechanical requirements of each site.

### 2.6. Bioactive Materials Necessary for Maxillofacial Bone Repair

Scaffolds are frequently treated on their surfaces. By incorporating different types of micro/nano surface materials (e.g., chitosan nanobeads [86]) into the scaffold design of osteogenic differentiation of stem cells, bone regeneration and bone tissue vascularization can be enhanced. Bone regeneration can be directly activated by targeting mitogen-activated protein kinase (MAPK) [87], signal transducer and activator of transcription (STAT) [88], and AKT signaling pathways [89,90]. The physical stimulation of cells by the surface topography of scaffolds can enhance bioactivity and biocompatibility, accelerating bone repair.

**Figure 5:**
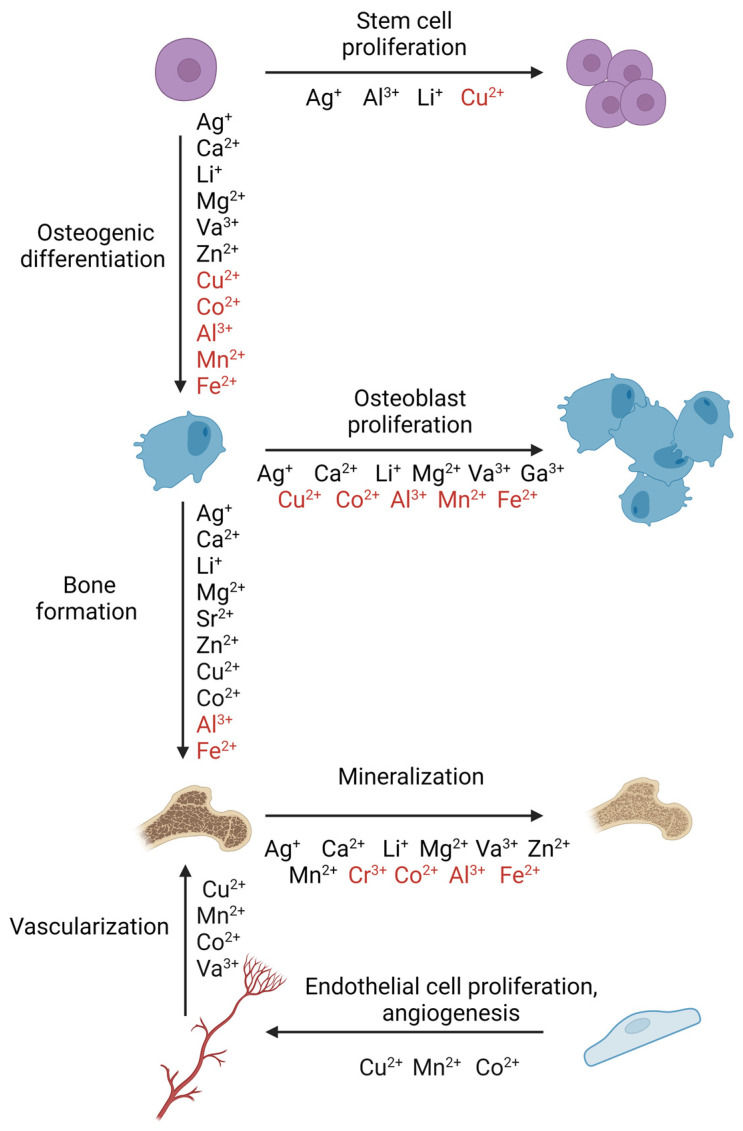
The importance of different bioactive ions during bone regeneration processes. Bioactive ions indicated in black improve the bone regeneration process, while bioactive ions indicated in red negatively influence bone regeneration [93].

In addition to direct activation of intracellular signaling in bone repair mechanisms, bioactive ions in bone regeneration are also applied to activate the bone regeneration process; 70% of the mass of bone is made up of minerals, 20% of collagen, and the remaining 10% is made up of various proteins, polysaccharides, and lipids [80]. The main minerals found in bones include magnesium, zinc, calcium, and strontium. These and several other bioactive ions play a central role in bone regeneration [91] (Figure 5) [92].

Magnesium is an essential element in bones and teeth. Magnesium complexes are biodegradable, and they can reduce bone resorption in the body [94,95]. Therefore, magnesium is important in treating bone defects [92]. Magnesium acts on the angiogenesis of the implanted tissue by the up-regulation of the secretion of the angiogenic factor VEGF [91,96,97]. In addition, osseointegration with the surrounding tissue can also be improved by magnesium [98,99].

Although strontium is not an essential element, it improves the osteoinductive properties of hydrogels, bioceramics, and metallic alloys [100,101,102]. Copper acts as an enzymatic cofactor in various enzymes influencing bone integrity, improves angiogenesis by up-regulation of VEGF expression [103], and induces tissue ingrowth [104,105]. The trace element zinc is essential for healthy bone development [106]. Hence, zinc ion-loaded scaffolds or biomaterials can successfully help bone regeneration processes and have antimicrobial and anti-inflammatory effects [106,107,108]. The above-listed examples show that the application of bioactive elements can potentially influence bone regeneration processes and can improve the bioactive properties of hydrogels, scaffolds, or metallic alloys.

The area where MRONJ develops is characteristic to an individual. Therefore, it must be ensured that personalized scaffolds are designed with the aid of CT scan-based clinical staging and scaffold design. The size of the necessary bone replacement, if discovered early, is frequently small, therefore allowing for the use of suitable hydrogel-based scaffold materials for tissue regeneration. These include thermal- or light-responsive scaffolds, chitosan hydrogel, and polymers that undergo irreversible gelation at 37 °C. Such hydrogels are suitable for clinical manipulation and can be combined with biocompatible photoinitiators for clinical application [109]. Various other hydrogels, including alginate hydrogel [110] and lipid hydrogel [111,112], have been experimented with to deliver drugs with the scaffolds. For similar reasons, magnetic-responsive scaffolds were also developed to increase the scaffold’s antimicrobial properties [113]. In a 3D-printed PLGA scaffold, magnetic nanoparticles [114,115], physiological endogenous electric fields, and electric conductivity are also suitable for antimicrobial effects stimulating bone regeneration [116,117] and restoration of the alveolar ridge [118]. The improved electrical conductivity can promote bone formation through external electrical stimulation and the piezoelectric effect in biomechanical environments [119].

Close connection between the graft and the recipient site is essential to ensure graft stability. To induce the cellularization of the scaffolds, additional factors like BMP-2, VEGF, and FGF are built into the material or to the surface (Table 4). Three-dimensionally printed PCL scaffolds with gradient pore sizes [54,96] for regeneration of large bone defects are well suited for the intricate geometry of the maxillofacial region [120]. A photocured HA-TCP scaffold made from a bio-ink containing polyfunctional acrylic resins and a photoinitiator was highly effective for osseointegration in bone defects in vivo [121].

While solid 3D scaffolds are essential for larger bone defects, they are sometimes augmented with injectable hydrogels and a variety of novel materials, including bioceramics [122], bioactive glass granules, or calcium phosphate cement (CPC) paste, to create extrudable and printable scaffold material [123]. Using such composite materials, the patient-specific implants result in tight contact between the graft and the defect. If the defect is minimal then it does not require invasive maxillofacial surgery [67,124]. Injectable biomaterials are not suitable for large bone defects and maxillofacial bone regeneration due to their lack of 3D stability and adequate porosity [82,125].

In theory, regenerating medication-induced maxillofacial bone damage should not pose a problem, as all technical issues have been resolved. However, creating a solid scaffold with the appropriate porosity and biological factors, along with the presence of ions that stimulate the growth and differentiation of mesenchymal stem cells (MSCs), still presents challenges. Specifically, there is a lack of vital information regarding the prediction of necrosis and the therapeutic drugs needed to expedite damage control in individual patients.

### 2.7. Drug Delivery in Maxillofacial Bone Regeneration

Numerous bioactive molecules have been identified that contribute to enhanced bone regeneration when used in conjunction with biomaterials. The primary medications may include antibiotics, anticancer drugs to reduce bone metastasis, or agents that promote bone regeneration following the treatment of MRONJ [126]. Information gained largely from animal studies is summarized in Table 5 [112,126,127,128,129,130,131,132,133].

## 3. Clinical Choices for Scaffold Materials for Maxillofacial Bone Regeneration and Current Clinical Trials

While, in theory, there are many options available for the therapy of medication-related osteonecrosis of the jaw (MRONJ), the clinical challenges are significant. MRONJ can often be asymptomatic for prolonged periods, making it difficult to predict who will develop this adverse reaction, how severe it will be, or when a patient might seek medical help. When symptoms do manifest, they typically begin with pain as the initial sign. In more advanced stages, MRONJ frequently presents with pain and purulent discharge from the exposed bone. The condition can also involve the teeth and gums, potentially leading to intraoral or extraoral fistulas.

It is clear that treatment selection depends on the stage of the disease. However, tissue engineering is not commonly incorporated into clinical practice. Additionally, choosing the appropriate therapy is complicated by the variety of scaffold materials available for maxillofacial bone regeneration. Based on clinical symptoms, the scaffold biomaterials must possess anti-inflammatory and antibacterial properties, which are critical in addressing the challenges posed by the bacteria-filled microenvironment of the oral cavity [134].

When personalized therapy is selected in MRONJ, several important factors are considered. These include the stage of MRONJ, whether the condition involves the mandibular or maxillary region, the need for surgical intervention, the characteristics of the bacterial microenvironment, the presence or absence of immune cells, the types of cytokines present, as well as the methods used for regenerative factors and the release of medication from scaffold materials. Currently, in limited clinical cases, various treatments are applied to promote tissue healing. These include BMP, autologous platelet concentrates (APCs), and leukocyte- and platelet-rich fibrin (L-PRF). These elements are often used in conjunction with VEGF and FGF to stimulate vascularization in methacrylated gelatin (Hep/GelMA) and in addition polycaprolactone (PCL) to provide the correct size of a structure. All of these elements are summarized in Table 6 [50,135,136,137,138,139].

If surgery becomes necessary, then scaffold implantation and induction of the bone tissue regeneration process becomes inevitable. Currently, artificial scaffold materials are mainly used in laboratories and tested in preclinical studies. An important aspect of their clinical application is matching the degradation rate of the scaffold with the rate of new tissue formation [67,140]. It is highly important that the formation of the bone is not affected by the metabolites of the degrading scaffold material; the absorption of residual bone engineering materials and the maintenance of augmented bone volume are inversely proportional and are strongly influenced by the solubility and acid resistance of the scaffold material [141]. It is important to highlight that in the maxillofacial region, the rates of bone remodeling in the maxilla and mandible are at least three times higher than those in the femur. As a result, the healing process in the maxillofacial region is faster. Therefore, the selection of scaffold formation must take this difference into account [142]. It is essential for scaffold degradation to align with rapid tissue formation, ensuring that degradation rates are coordinated with the release of drugs, growth factors, and biomolecules [22,143]. It is important to emphasize that suitable MSC selection is crucial to prevent rejection and ensure proper differentiation in maxillofacial surgery.

## 4. Prospects for MRONJ Therapy

The field of biomaterial sciences and tissue engineering has made remarkable advancements in the study of bone regeneration. It has been discovered that specific BMSCs are particularly effective in maxillofacial bone regeneration, differing from those required for long bones. While existing treatments for osteoporosis and bone metastasis can result in osteonecrosis in maxillofacial bones, the underlying causes of this condition and effective treatments need further studies.

BPs bind to calcium surfaces and selectively target bone minerals, effectively inhibiting osteoclast-mediated bone resorption. However, a different approach is necessary to reduce the concentration of BPs in maxillofacial bones and prevent necrosis. Another potential solution is to administer medications beyond antibiotics to counteract the necrotic process triggered by BPs in this specific area.

The personalized approach and the choice of tissue engineering materials in MRONJ therapy is influenced by the size and shape of the lesion, the exposure of the bone to pressure, and the involvement of surrounding tissue, which determine the procedure to be used.

Understanding the process of MRONJ is crucial to comprehend the entire process of osteonecrosis and the effects of medications that trigger MRONJ. Once potential mechanisms are identified, it may be possible to specifically target the disease process and progression in future treatments.

Currently, comprehensive studies on MRONJ are lacking and further analysis is needed in the following fields:

1. A detailed analysis of the causes of MRONJ.
2. An exploration of the genetic and clinical factors that may increase a patient’s susceptibility to MRONJ.
3. Extensive studies are needed to investigate how micro-RNAs (miRNAs) in saliva and blood regulate maxillofacial bone destruction, as well as the role of circular RNAs in controlling miRNAs that contribute to MRONJ in specific patients.
4. An examination of how an individual’s molecular background can lead to adverse reactions to specific medications.

Our manuscript has highlighted a broad range of novel methods to potentially treat MRONJ. Determining who is suitable for therapy using scaffold- or hydrogel-based tissue engineering methodologies is challenging. The information listed above is essential to initiate safe clinical trials in patients.

However, to collect vitally important information, apart from the above-mentioned, clinical studies need to be investigated in combination with in vitro analysis of clinical information using in vitro maxillofacial bone models. Without such studies, it is difficult to find reliable directions and recommendations to begin clinical trials for effective and potentially preventive treatment of MRONJ.

Current clinical studies exploring stem cell therapy [144] and the effects of ozone infiltration in randomized clinical trials [145] have recently been reported. While it appears that many clinical trials have been performed, products of tissue engineering-based reports are seriously limited. Most studies are reporting a subsegment of using various biomaterials for treating maxillofacial bone defects or a variety of stem cells, but only with short follow-up periods, small sample sizes, and unspecified differences in treatment protocols [146]. Nevertheless, several meta-analyses of MRONJ have been reported in recent years [147], but most publications do not refer to tissue engineering methodologies. To bolster the evidence base, high-quality in vitro human maxillofacial bone models and clinical studies are urgently needed to translate preclinical research into practical clinical applications. Factors such as manufacturing, cost, and the consistency of these innovative biomaterials from batch to batch need to be carefully considered.

## Figures and Tables

**Table 1:** Classification of pharmaceutical agents that can induce osteonecrosis of the jaw. The table describes the various indications and mechanisms of action of the main drugs classified into drug categories and subcategories (receptor activator of NF-kappaB ligand (RANKL), vascular endothelial growth factor (VEGF), adenosine triphosphate (ATP), platelet-derived growth factor (PDGF), mammalian target of rapamycin (mTOR), metastatic colorectal cancer (mCRC), metastatic renal cell carcinoma (mRCC), non-small cell lung cancer (NSCLC), gastrointestinal tract (GI), hepatocellular carcinoma (HCC), lymphangioleiomyomatosis (LAM), angiomyolipoma (AML)).

Pharmaceutical Classification	Subcategory	Mechanism of Action	Molecule	Indication
**Antiresorptive agents**	Bisphosphonates	Inhibit calcification Inhibit hydroxyapatite breakdown Bone resorption suppression Osteoblast and osteocyte apoptosis restriction	Alendronate	Osteoporosis
			Ibandronate	Osteoporosis
			Neridronate	Osteogenesis imperfecta
			Pamidronate	Bone metastasis
			Risedronate	Osteoporosis
			Zolendronate	Osteoporosis, Bone metastasis
	RANKL-inhibitor	Inhibition of the development and activity of osteoclasts Decrease bone resorption Increasing bone density	Denosumab	Osteoporosis, Bone metastasis
**Antiangiogenic drugs**	VEGF-trap	Soluble fusion protein, with a high affinity to VEGF Blocks VEGF signaling	Aflibercept	mCRC, Macular degeneration, Retinopathy
	Anti-VEGF monoclonal antibodies	Blocks VEGF signaling	Bevacizumab	mCRC, mRCC, NSCLC, Glioblasoma
	Tyrosine-kinase inhibitory small molecules	Binds to the ATP- binding catalytic site of the tyrosine kinase domain of VEGFRs Blocks the intracellular signaling of VEGFR	Sunitib	GI stromal tumors, RCC
			Sorafenib	HCC, RCC
			Cabozantinib	mRCR
	mTOR inhibitor	Decreases the production of VEGF and PDGF	Rapamycin	Organ transplantation, LAM, AML

**Table 2:** Summary of MRONJ stages according to various classifications. The table summarizes the clinical symptoms and outcomes associated with the various stages of MRONJ (osteoradionecrosis (ORNJ), American Association of Oral and Maxillofacial Surgeons (AAOMS)).

Stages of MRONJ (AAOMS)	Exposed or Necrotic Bones	History and Clinical Findings	Notani et al. Classification for ORNJ [16]	Clinical Features
**Stage 0**	No clinical evidence	Non-specific clinical and radiographical findings	**Type I**	ORNJ confined to dentoalveolar bone
**Stage 1**	Exposed and necrotic bone or fistulae that probes to bone	Asymptomatic with no evidence of infection	**Type II**	ORNJ limited to dentoalveolar bone or mandible above the inferior canal or both
**Stage 2**	Exposed and necrotic bone or fistulae that probes to bone	Associated with infection, pain and erythema in the region of the exposed bones with or without purulent damage	**Type III**	ORNJ involving the mandible below the inferior dental canal or pathological fracture or skin fistula
**Stage 3**	Exposed and necrotic bone or fistulae that probes to bone	Pain, infection and one or more of the following: Exposed necrotic bone extending beyond the alveolar bone region resulting in pathological fracture Extraoral fistula, Oro-nasal or oro-antral communication Lytic changes extending to the lower border of the mandible or sinus floor	**Epstein et.al classification for ORNJ [17]**	**Clinical features**
			**Type I**	Resolved, healed: No pathologic fracture Pathologic fracture
			**Type II**	Chronic persistent (nonprogressive): No pathologic fracture Pathologic fracture
			**Type III**	Active progressive: No pathologic fracture Pathologic fracture

**Table 3:** Summary of specific signals for osteogenesis and angiogenesis. The molecular factors listed in the table are crucial in long bone and/or maxillofacial bone and/or cartilage formation process. They act in different ways: they can influence bone vascularization–vascular endothelial growth factor (VEGF), bone differentiation, mesenchymal tissue formation, fibroblast growth factor (FGF), or have a complex developmental effect (WNT/β-catenin). Collagen type 1 alpha 1 chain (Col1a1) helps in bone extracellular matrix formation, while collagen type 2 alpha 1 chain (Col2a1) Col2a1 affects cartilage tissue development (Col1a1, Col2a1, SRY-Box Transcription Factor 9 (Sox9), bone morphogenic protein (BMP), vascular endothelial growth factor (VEGF), fibroblast growth factor (FGF), not applicable (N/A)).

Molecule	Role	Maxillofacial Bones	Long Bones	Cartilage	Reference
**Col1a1**	Extracellular matrix of bone	+	+	-	[31]
**Col2a1**	Cartiligous template	-	-	+	[32]
**Sox9**	Binds to Col2a1	-	-	+	[33]
**WNT/ß-catenin**	Complex developmental effect	+	+	-	[30,34]
**BMP2**	Induces bone differentiation	+	+	+	[35,36]
**VEGF**	Vascularisation	N/A	N/A	N/A	[37,39]
**FGF**	Mesenchymal tissue formation	N/A	N/A	N/A	[38]

**Table 4:** Materials and manufacturing technologies for scaffolds: The table represents various biodegradable (PCL, PLGA) and non-biodegradable (acrylic resin) materials in preclinical applications characterized by their processing technology, optimal pore size, and additive materials (poly-caprolactone (PCL), ß-tricalcium-phosphate (ß-TCP), nano-hydroxyapatite (nHA), hydroxyapatite (HA), thermoplastic polyurethane (TPU), fused deposition modelling (FDM), stereolithography (SLA)).

Base Material	Additive Material	Processing Technology	Pore Size	Preclinical Use Case	Reference
**PCL**	ß-TCP	Material extrusion (Multi-head Deposition System)	500 µm	Maxillary bone regeneration	[51]
			400 µm	Mandibular bone regeneration	[53]
		FDM	515 µm	Bone regeneration	[52]
	nHA	FDM	300 µm/500 µm	Osteogenic performance improvement	[54]
	–	Melt Electrospinning Writing	225 µm/500 µm	Oral and maxillofacial bone regeneration (in vitro)	[50]
**PLGA**	ß-TCP or ß ß-TCP+TPU	Solvent-based 3D printing	60 µm 130 µm	Bone tissue engineering purposes (in vitro)	[56]
	ß-TCP +Poly(dopamine) coat	Solvent-based 3D printing	~500 µm	Bone tissue engineering	[59]
	HA	FDM	~350 µm	Bone tissue engineering	[60]
		Bioprinting	~400–450 µm	Mandibular bone regeneration	[55]
**PLGA+PCL**	ß-TCP	Heating and compression	–	Bone tissue engineering	[58]
	HA	Material extrusion (in-house development)	~200–400 µm	Bone tissue repair	[57]
**Acrylic resin**	HA and HA60-TCP40	SLA	–	Bone tissue regeneration	[61]

**Table 5:** Potential drug delivery applications in maxillofacial bone regeneration. The table summarizes the two main drug delivery options: scaffold-based and extracellular vesicle/nanoparticle-based drug delivery methods. It presents their active ingredients, characteristic scaffold or carrier materials, and their roles in the bone regeneration process (bone morphogenic protein 2 (BMP2), AdipoRon (APR), bone marrow stem cells (BMSC), messenger RNA (mRNA), micro RNA (miRNA), extracellular vesicle (EV), poly-lactic acid (PLA), tricalcium phosphate (ß-TCP), poly-caprolactone (PCL), polyethylene glycol (PEG), hydroxyapatite (HA), poly(lactide-co-glycolide) (PLGA)).

	Active Ingredients	Role	Scaffold Material	Reference
**Scaffold-based drug delivery**	Antibiotics metronidazole and ornidazol	Eliminate the growth of anaerobic organisms	Hydrogels with special properties (HA, PEG, Alginate, Chitosan)	[126,127]
	Anti-inflammatory drugs: curcumin	Inhibits NF-kappa signalling	Hyaluronic acid sponge loaded with curcumin	[127]
	Antimicrobial peptides, Silver, Copper	Induction of oxidative stress		[129]
	Growth Factor erythropoietin	Alveolar ridge regeneration, improved bone and blood vessel formation	Collagen and gelatin sponges, hydrogels	[112,130]
	Antibiotics, BMP2	Inhibition of bacterial growth, induction of cellular infiltration	PLA scaffold coated with polyelectrolyte film for BMP2 delivery	[131]
	BMP2, Tea polyphenols (TP), AdipoRon (APR)	Induction of cellular infiltration	Core-shell structure	[132,133]
**Extracellular vesicles (EV) and nanoparticle drug delivery**	BMP2 polydopamine-heparin nanoparticles	Induction of cellular infiltration	Nanoparticles loaded onto a novel hydrogel scaffold	[129]
	BMSC or cell aggregates loaded on scaffold	Induction of cellular growth and bone formation	Bioceramics or hydrogels	
	BMP2, mRNA, miRNA or any other drug	Induction of cellular infiltration and bone differentiation	Stem cell-derived EV or artificial EV filled with molecules	

**Table 6:** The table summarizes the potential therapeutic interventions in various MRONJ stages. The MRONJ stage highly influences the regenerative potential of the bone, and therefore the applicable scaffold materials, molecular signaling molecules, and medications as well (bone morphogenic proteins (BMP), parathyroid hormone, photobiomodulation (PBM), autologous platelet concentrates (APCs), leukocyte- and platelet-rich fibrin (L-PRF), vascular endothelial growth factor (VEGF), fibroblast growth factor (FGF), methacrylated gelatin (Hep/GelMA), polycaprolactone (PCL), not applicable (N/A)).

MRONJ Stages	Surgery	Scaffold Material	Pore Size	Factors	Cell Type	Medication	Reference
**0**	N/A	N/A or hydrogel (Hep/GelMA)	N/A	N/A	N/A	Systematic antibiotics and/or antimicrobial rinse	[135,136]
**I**	N/A	Hydrogel (Hep/GelMA)	N/A	BMPs, PTH, PBM, APCs, L-RPF	N/A	Systematic antibiotics and/or antimicrobial rinse, adjuvant hyperbaric oxigen	[137,138,139]
**II**	N/A (Surgery)	Hydrogel (Hep/GelMA)	N/A	PRF+BMP, L-PRF	N/A	Systematic antibiotics and/or antimicrobial rinse	[137]
**III**	Surgery	Bioactive PCL	225 µm and 500 µm	BMP2, VEGF, FGF, APCs, L-PRF	BMSC	Systematic antibiotics antimicrobial peptides and metal ions	[50]

## Data Availability

No new data were created or analyzed in this study. Data sharing is not applicable to this article.
